# Long-Term Survival After Intravenous Thrombolysis for Ischemic Stroke: A Propensity Score-Matched Cohort With up to 10-Year Follow-Up

**DOI:** 10.1161/STROKEAHA.117.019889

**Published:** 2018-02-12

**Authors:** Walter Muruet, Anthony Rudd, Charles D.A. Wolfe, Abdel Douiri

**Affiliations:** From the School of Population Health & Environmental Sciences, King’s College London, United Kingdom (W.M., A.R., C.D.A.W., A.D.); NIHR Comprehensive Biomedical Research Center, Guy’s and St Thomas’ NHS Foundation Trust, and King’s College London, United Kingdom (A.R., C.D.A.W., A.D.); and National Institute of Health Research Collaboration for Leadership in Applied Health Research and Care (CLAHRC) South London, United Kingdom (C.D.A.W.).

**Keywords:** activities of daily living, prognosis, propensity score, risk, stroke

## Abstract

**Background and Purpose—:**

Intravenous thrombolysis with alteplase is one of the few approved treatments for acute ischemic stroke; nevertheless, little is known about its long-term effects on survival and recovery because clinical trials follow-up times are limited.

**Methods—:**

Patients registered between January 2005 and December 2015, to the population-based South London Stroke Register of first-ever strokes. Propensity score was used to match thrombolyzed and control cases to a 1:2 ratio by demographical and clinical covariates. The primary outcome was survival up to 10 years using Kaplan–Meier estimates, Cox proportional hazards, and restricted mean survival time. Secondary outcomes included stroke recurrence and functional status (Barthel Index and Frenchay Activities Index scores) at 5 years.

**Results—:**

From 2052 ischemic strokes, 246 treated patients were matched to 492 controls. Median follow-up time 5.45 years (interquartile range, 4.56). Survival was higher in the treatment group (median, 5.72 years) compared with control group (4.98 years, stratified log-rank test <0.001). The number needed to treat to prevent 1 death at 5 years was 12 and 20 at 10 years. After Cox regression analysis, thrombolysis reduced risk of mortality by 37% (hazard ratio, 0.63; 95% confidence interval [CI], 0.48–0.82) at 10 years; however, after introducing a multiplicative interaction term into the model, mortality risk reduction was 42% (hazard ratio, 0.58; 95% CI, 0.40–0.82) at 10 years for those arriving within 3 hours to the hospital. On average, in a 10-year period, treated patients lived 1 year longer than controls. At 5 years, thrombolysis was associated with independence (Barthel Index≥90; odds ratio, 3.76; 95% CI, 1.22–13.34) and increased odds of a higher Frenchay Activities Index (proportional odds ratio, 2.37; 95% CI, 1.16–4.91). There was no difference in stroke recurrence.

**Conclusions—:**

Thrombolysis with intravenous alteplase is associated with improved long-term survival and functional status after ischemic stroke.

## Abstract 2

Supplemental Digital Content is available in the text.

Evidence from randomized controlled trials and meta-analysis shows improved functional outcomes for acute ischemic stroke patients receiving intravenous tissue thrombolysis with the recombinant tissue-type plasminogen activator alteplase.^1–3^ However, it is still unclear whether intravenous thrombolysis has any effect on mortality, particularly in the long-term. This has led to some concerns of whether the early risks associated with thrombolysis (eg, intracranial hemorrhage) translate into better prognosis over time.^4^ Furthermore, most of the studies that have examined differences in outcomes between groups have had a limited follow-up time (5–17). Currently, information available about effects on survival after intravenous thrombolysis come from 2 randomized clinical trials and 3 observational studies.^5–9^ The National Institute of Neurological Disorders and Stroke Recombinant Tissue Plasminogen Activator Stroke Study assessed mortality at 12 months, without finding any significant difference between the 2 study arms.^5^ More recently, a study using participants from the IST-3 (Third International Stroke Trial) found a small reduction in 3-year mortality in the treatment arm which was nonsignificant for all study subjects but only for those who survived the first week.^6^ However, the IST-3 trial randomized patients who did not meet current eligibility criteria for intravenous thrombolysis with alteplase in standard practice (ie, patients after 4.5 hours from stroke onset). Three observational studies have examined long-term outcomes of intravenous alteplase; however, 2^7,8^ are limited by the lack of a comparison group. The third study,^9^ and the only other propensity score-matched study on intravenous alteplase found a 34% decrease in mortality for treated stroke patients in Denmark. However, this study had a limited median follow-up of 1.4 years and did not examine differences in activities of daily living between groups.

In this study, we use a propensity score-matched cohort study design to determine whether thrombolysis with intravenous alteplase, as given in standard daily clinical practice in the United Kingdom,^10^ improves long-term survival up to 10 years after an acute ischemic stroke. Propensity score methods are tools for the analysis of observational studies that allow reducing the effect of the confounding that can occur because differences in the distribution of baseline characteristics and allow to replicate the measures of effect commonly reported in randomized clinical trials.^11^ As secondary outcomes, we examine whether the benefits in functional status, as assessed by the Barthel Index (BI) and Frenchay Activities Index (FAI), persist at 5 years after a stroke, as well as if stroke recurrence is affected by intravenous alteplase.

## Methods

The data that support the findings of this study are available from the corresponding author on reasonable request.

### Study Design

The South London Stroke Register (SLSR) is an ongoing, prospective, population-based, stroke register. The SLSR started in January 1995 and documents all first-ever confirmed strokes (according to the World Health Organization Criteria^12^) in patients of all ages for an inner area of South London that includes 22 electoral wards in the Boroughs of Lambeth and Southwark.^13^ The total source population of the SLSR area is 357 308 inhabitants, as estimated in the 2011 census and comprises a distinctly multiethnic population with a significant proportion of black Caribbean and African residents.^14^

### Case Ascertainment

All patients with a suspected diagnosis of first-ever stroke documented from hospital- and community-based sources were investigated for study eligibility.^12^ Completeness of case ascertainment has been estimated at 88% by a multinomial logit capture-recapture model using the methods described elsewhere.^15^ A more thorough discussion of the methods used to maximize completeness of case ascertainment is available elsewhere.^12,16^

### Data Collection

Specially trained study nurses and field workers collected all data prospectively. Patients were examined within 48 hours of referral to SLSR when possible.^12^ A study stroke physician verified the diagnosis of stroke and classified the cases according to the modified TOAST (Trial of ORG 10172 in Acute Stroke Treatment)^17^ and the Oxfordshire Community Stroke Project subtype.^18^ The Oxfordshire Community Stroke Project subtype, which was used to calculate the propensity score, consists of 4 defined subgroups total anterior circulation infarcts, partial anterior circulation infarcts, lacunar infarcts, and posterior circulation infarcts.

Age at stroke was calculated as the difference between the date of birth and date of symptoms onset; ethnicity was self-reported by the patient and then collated into 1 of the 3 main categories according to the UK 2001 census (White, Black, and Other).^13^

Vascular risk factors before stroke (self-reported and from medical notes) were collected, including smoking, hypertension, diabetes mellitus, atrial fibrillation, ischemic heart disease (angina pectoris or myocardial infarction), peripheral vascular disease, and history of transient ischemic attack. Information on thrombolysis was collected from medical charts from 2005 onwards. The window for thrombolysis with intravenous alteplase was up to 3 hours after symptoms’ onset at the start of the study period and then extended up to 4.5 hours from 2009 onwards.

The BI^19^ was used to assess functional status previous to stroke as well as at each follow-up visit. A cutoff of BI≥90 was used to reflect functional independence.^20^ The National Institutes of Health Stroke Scale (NIHSS) score was used to assess stroke severity^21^ during the acute phase. The degree of neurological deficit was classified according to the total NIHSS score as follows minor (NIHSS, 1–4), moderate (5–15), moderate-severe (16–20), and severe (≥21). The FAI is a frequently used measurement of Extended Activities of Daily Living in stroke and was assessed by trained fieldworker during follow-up visits.^22^ Follow-up visits were performed at 3 months, 1 year, and annually thereafter. Survival was ascertained by follow-up, which included contacting next of kin if the participant was unreachable, and by checking with the office of National Statistics. Because the registration of deaths in the United Kingdom is complete, all patients with no death record at the day of censoring were assumed to be alive.

### Study Population

The study included patients recruited into the SLSR from 2005 to 2015, inclusive. Survival time was censored at the December 31, 2015. All patients receiving intravenous thrombolysis with complete data for matching variables (see below) were included in the analysis. None of the participants in this study were treated with intra-arterial thrombolysis or mechanical clot retrieval.

### Ethical Approval

The study was approved by the ethics committees of Guy’s and St Thomas’ Hospital Trust, King’s College Hospital, Queen’s Square, and Westminster Hospital. Informed consent from patients or assent from next of kin was obtained for all participants for their inclusion into the South London Stroke Register.

### Propensity Score Matching

**Figure 1.**
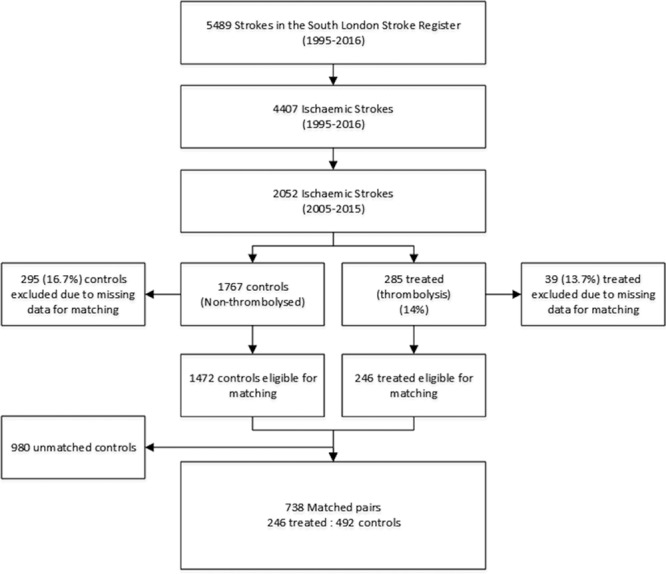
Data attrition flowchart.

Propensity scores were calculated for each patient based on a multivariable logistic regression model. This model included demographic variables (age, sex, and ethnicity), prestroke number of vascular risk factors, functional status previous to stroke (BI), stroke severity (NIHSS), stroke Oxfordshire Community Stroke Project subtype,^23^ and year of stroke. Figure I in the online-only Data Supplement shows the substantial overlap in propensity score distributions between both groups; this suggests a large area of common support for the eligible participants. We matched treated participants with controls in a 1:2 ratio using a greedy nearest neighbor method.^24^ Figure 1 presents the flowchart of participant selection and propensity score-matched set construction. The overall quality of the matched sample was assessed by comparing the standardized difference of means and the ratio of the variances between the propensity scores of both groups as well as by graphically inspecting the propensity scores between groups. Furthermore, we evaluated the balance between individual covariates between groups in the matched sample.

### Statistical Analysis

Descriptive data are expressed in percentages, mean±SD or median and interquartile range (IQR) as appropriate. The primary outcome of this study was survival up to 10 years after the date of first-ever acute ischemic stroke; we report Kaplan–Meier survival estimates and the difference between survival curves tested using the log-rank test stratified to matched sets. We obtained the adjusted hazard ratio (HR) from a Cox regression model of proportional hazards with robust variance estimator. The Cox model was developed by iteratively adding clinical relevant variables to a model including only treatment arm (ie, treated or control) regressed by the propensity score, and used a log-likelihood test to evaluate whether the addition of the new predictor improved the fit of the previous model. To examine whether onset-to-arrival time modified the effect of alteplase, we then tested if a multiplicative interaction term between treatment with alteplase and arrival within 3 hours further improved the fit our model. Because we expected, based on the reviewed literature,^6^ that the proportionality of the hazards assumption would be violated, we further assessed survival time after stroke by comparing the restricted mean survival time (RMST) between groups. We then adjusted the RMST for the same covariates used in the Cox model with an analysis of covariance.^25^ A similar method was used to examine the difference in stroke recurrence between groups. Independence at 5-year BI (≥90) and FAI scores at 5-year follow-up were compared between groups and adjusted for age, sex, ethnicity, prestroke BI, acute phase NIHSS, and stroke subtype by performing multiple regression analysis. We conducted a sensitivity analysis with multiple imputation to examine how robust our results were to missing data. All analyses were performed using R^26^ version 3.2.2 (2015) on R-Studio^27^ version 1.0.136.

## Results

A total of 2052 patients with their first-ever ischemic stroke were recruited between the January 1, 2005 and the December 31, 2015; 285 (13.9%) of these patients received intravenous thrombolysis with alteplase. From the total recruited, 334 (16.3%) had missing data for at least 1 of the variables used to calculate the propensity score and thus had to be excluded. Of the 1718 remaining subjects, we paired 246 treated patients with 492 controls (Figure 1). None of these patients received intra-arterial thrombolysis or underwent thrombectomy. Measures of balance diagnosis^28^ indicated that the sample was adequately matched, with a standardized difference of the means of propensity scores between groups of 0.14 (good balance<0.25) and a ratio of variances of propensity scores of 1.27 (good balance between 0.5–2). A comparison of the baseline characteristics further supports the good balance of our matched sample (Table 1; Table I in the online-only Data Supplement).

**Table 1.**
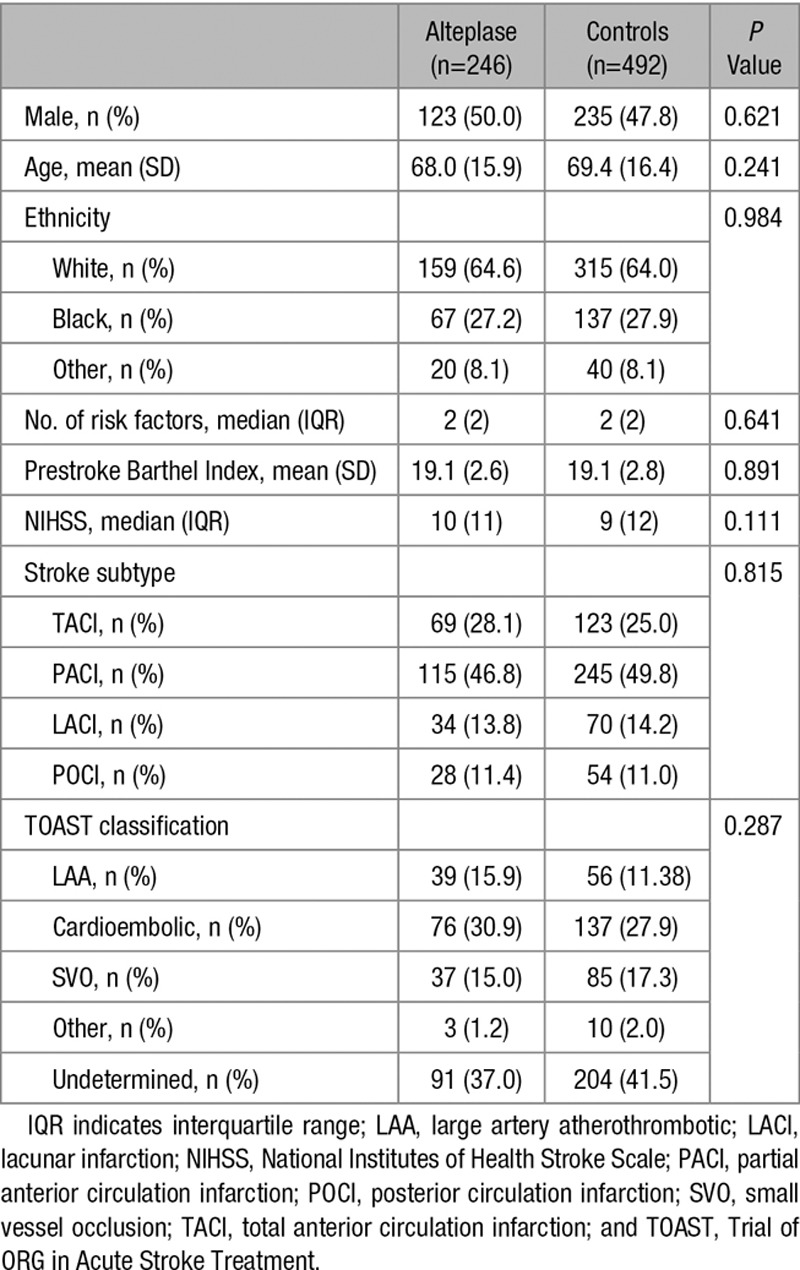
Baseline Characteristics

The median follow-up time was 5.45 years (IQR=4.56; range, 0–10 years), and a total of 344 (46.6%) patients died during the study period.

### Primary Outcome: Survival up to 10 Years

**Figure 2.**
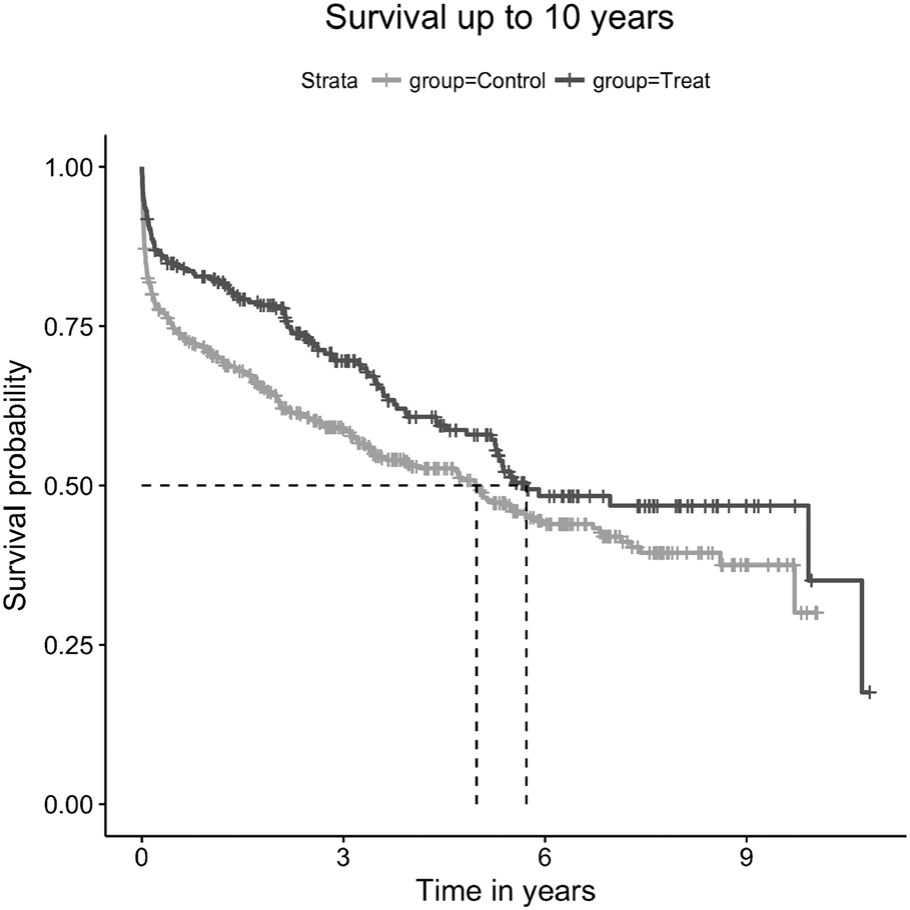
Survival curves for intravenous alteplase treated group (darker) and control group (lighter) groups. Median follow-up time 5.45 years. Median survival for treated group 5.72 years. Median survival for control group 4.98 years. Stratified log-rank test: *P*<0.001.

The Kaplan–Meier estimate shows a higher survival for patients treated with intravenous alteplase than for those in the control group at 5 and 10 years (Figure 2; log-rank test stratified by sets <0.001 for both). The median survival time for the treated group was 5.72 and 4.98 years for the control group. The absolute risk reduction at 5 years was 8.33% (95% confidence interval [CI], 8.19–8.47; number needed to treat, 12) and 5.07% (95% CI, 4.92–5.22) at 10 years (number needed to treat, 20).

The unadjusted HR shows a 19% (HR, 0.81; 95% CI, 0.70–0.92) and 28% (HR, 0.72; 95% CI, 0.57–0.91) decrease in mortality risk for the treated group at 5 and 10 years, respectively. After adjusting for age, prestroke BI, prestroke use of anticoagulants, NIHSS during the acute phase, and poststroke treatment with antiplatelets, thrombolysis with intravenous alteplase was associated with a 28% (HR, 0.72; 95% CI, 0.60–0.87) decrease in mortality at 5 years and 37% (HR, 0.63; 95% CI, 0.48–0.82) at 10 years (Table II in the online-only Data Supplement). After including a multiplicative interaction term between thrombolysis with intravenous alteplase and arrival to the hospital within 3 hours, mortality reduction for those treated earlier was 32% (HR, 0.67; 95% CI, 0.52–0.88) at 5 years and 42% (HR, 0.58; 95% CI, 0.40–0.82) at 10 years (Table 2; Figure II in the online-only Data Supplement).

**Table 2.**
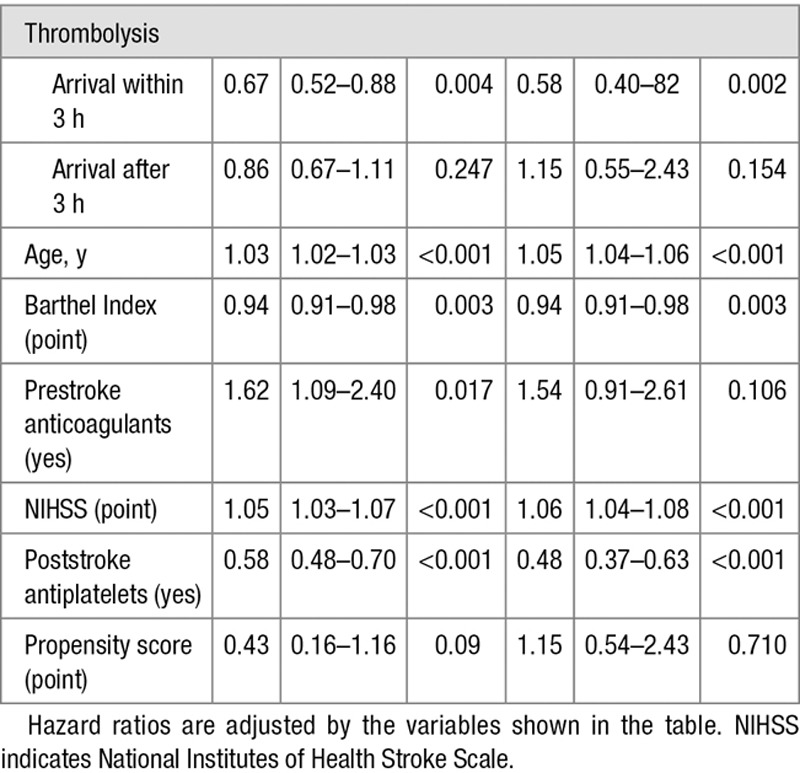
Multivariable Cox Regression on Survival Including Interaction Term

Visual and formal testing revealed hazards to be nonproportional (*P*<0.0001) for the whole duration of the follow-up. To account for this, we calculated the RMST for each group. Patients receiving intravenous alteplase had an RMST of 6.06 years, whereas the control group RMST was 5.18 years. The estimated difference in RMST between groups was 0.88 years (95% CI, 0.18–1.59; *P*=0.015) over a 10-year follow-up period. After adjustment for the same covariates used in the Cox proportional hazards model, the estimated difference between RMST was 1.04 years (95% CI, 0.17–1.91; *P*=0.02).

### Secondary Outcomes: Functional Status at 5 Years

Thrombolysis with intravenous alteplase was associated with improved functional status. After adjusting for age, sex, ethnicity, prestroke BI, acute phase NIHSS, and stroke subtype, treatment was significantly associated with independence (BI≥90) at discharge (odds ratio, 2.01; 95% CI, 1.27–3.20) and at 5 years (odds ratio, 3.76; 95% CI, 1.22–13.34). Intravenous alteplase was also associated with increased odds of higher FAI score (proportional odds ratio, 2.37; 95% CI, 1.16–4.91) at 5 years. There was no difference in stroke recurrence between groups.

## Discussion

### Key Findings

**Figure 3.**
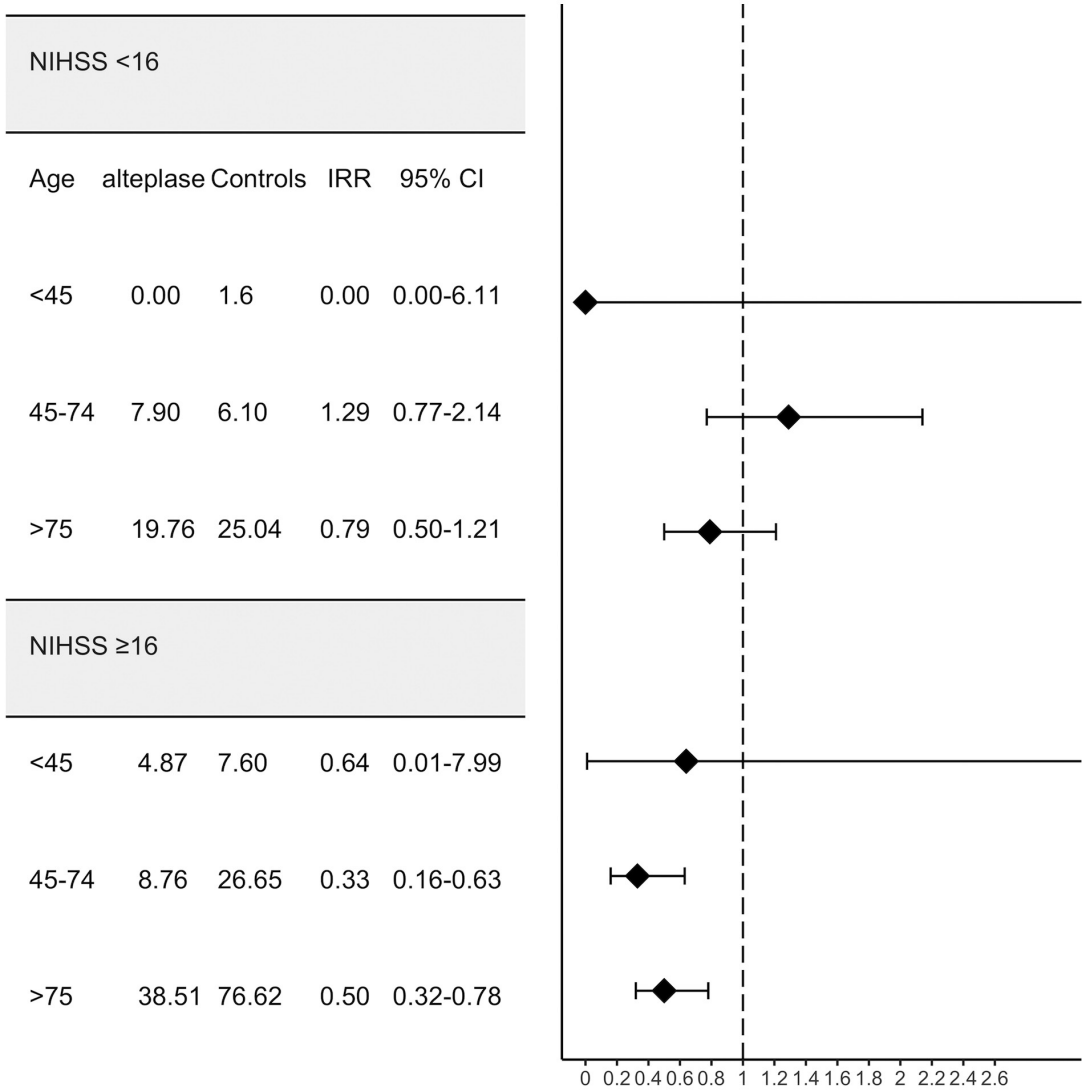
Forest plot showing the point estimates incidence rate ratio (IRR) with their respective CI for mortality per 100 person-year stratified by age and National Institutes of Health Stroke Scale (NIHSS) group. Median (interquartile range) NIHSS<16 group was 7 (5); NIHSS≥16 group was 21 (6). CI indicates confidence interval.

In this study, which to our knowledge has the longest median follow-up time in the published literature, we found evidence in a real-world setting of reduced mortality after thrombolysis with intravenous alteplase. Our findings show that on average, and over a 10-year period, a patient treated with thrombolysis lives around 1 year longer than a similar nonthrombolyzed patient after adjustment for age, sex, prestroke BI, prestroke treatment with anticoagulants, acute phase NIHSS score, and poststroke treatment with antiplatelets. Our data shows that the number needed to treat to prevent 1 death in 5 years is equal to 12 patients and 20 to prevent 1 death at 10 years. These results complement and expand on previous data reported by the Danish Stroke Register^9^ and the subanalysis of the IST-3 trial^6^ by demonstrating that the benefits of thrombolysis in survival are appreciable even after a period as long as 10 years poststroke and the improved functional outcomes are still perceivable at 5 years. Furthermore, our findings also suggest that the observed benefit in survival is seemingly driven by older patients and those with NIHSS≥16 (moderate-severe and severe strokes; Figure 3).

Compared with the Danish nationwide register study,^9^ our study had a slightly older mean age (treated, controls; SLSR, 68.0, 69.4 versus Danish Register, 65.8, 66.5) a significantly longer median follow-up time (5.45 versus 1.4 years), and consequently a higher proportion of deaths (46.6% compared with 14.7%). Furthermore, our study included a higher proportion of moderate (48.4%, 56.7% versus 33.8%, 33.9%) and severe strokes (16.7%, 15.2% versus 7.7%, 7.9%), a comparable proportion of moderate-severe strokes (17.5%, 12.2% versus 14.9%, 15.0%) and significantly fewer minor strokes (17.5%, 15.9% versus 41.4%, 41.5%) according to the scale used by each study (NIHSS versus Scandinavian Stroke Scale).The median stroke severity was also slightly higher in our cohort (10, 9 versus 8, 8, converted from Scandinavian Stroke Scale to NIHSS using the formula found elsewhere^29^). Our population also had a higher prevalence of hypertension (64.2%, 65.7% versus 50.9%, 47.8%), and diabetes mellitus (20.3%, 21.1% versus 9.6%, 12.6%; Table I in the online-only Data Supplement). Additionally, our population was comprised of first-ever strokes only. Despite these differences our adjusted HR for the whole duration of the study, without time to arrival interaction term, are similar (0.63 [95% CI, 0.48–0.82] versus 0.66 [95% CI, 0.49–0.88]) suggesting the benefits from intravenous alteplase are generalizable across populations.

The precise mechanism or mechanisms by which thrombolysis improves survival are unknown, although there is an evidence thrombolysis decreases infarct size^30^ and reduces the risk of readmissions because of pneumonia,^31^ although the impact on other factors is still unclear. Nevertheless, previous studies have shown that good functional outcomes in the short term (ie, within 6 months) are associated with improved long-term survival, in part because of fewer complications and more independence.^9,32,33^ In our cohort, patients in the treatment group had overall better scores in BI and FAI even at 5-years poststroke after adjustment, further suggesting the association between functional status and survival. Furthermore, better BI scores have been strongly associated with quality of life^34^ which means that the improved survival seen with alteplase is also accompanied by improved quality of life.

### Strengths and Weaknesses

The main strengths of this study include a long follow-up time (up to 10 years, median 5.45 year), a per protocol, prospectively collected data set with a wide range of variables that allowed a good balance in baseline variables between groups, an ethnically diverse study population of a well-defined area with a near-complete recording of deaths, achieved by linking with the Office of National Statistics and follow-up by the register fieldworkers. Additionally, we provide the adjusted difference in RMST, a summary statistic which has been shown to better estimate time-to-event than the HR when the proportionality of the hazards assumption is not met.^25^ Furthermore, the difference in RMST can be straightforwardly interpreted in clinical settings by both the treating physician, the patients and their next of kin. The main limitation of this study lies in its design as an observational rather than experimental study. However, we have used propensity score matching to reduce potential bias and strengthen our reported effect estimates.^35^ Additionally, we matched every treated patient with a similar control and included the calculated propensity score into the multivariable analysis (double propensity score adjustment), thus reducing as much as possible confounding because of incomplete matching and residual confounding. Another limitation is the effect of missing data in the propensity score matching as well as the analysis. This limitation is common to all studies, particularly those with long follow-up times. Nevertheless, a sensitivity analysis demonstrated that our results were robust to the influence of missing data. Additional limitations include that the SLSR does not collect information about the time of thrombolysis and that the modified Rankin Scale score is not available for follow-ups before 2014. However, although it is not possible for us to calculate the onset-to-treatment time, we have used onset-to-arrival times as a proxy instead; this value is not only correlated with the time from onset to treatment, but also is available for nontreated patients, and thus, it could be fitted into the Cox model. Although we do not have enough data on the modified Rankin Scale score at 5 years, we have used the BI with a threshold of ≥90 to define independence; using this cutoff point has been shown to be comparable to a modified Rankin Scale score of ≤2^20^.

### Implications for Clinical Practice

Despite the amount of evidence provided by clinical trials demonstrating that thrombolysis with alteplase improves functional outcomes in ischemic strokes at 1 year, the adoption of thrombolysis has been slow by many centers. Although the reasons for this are multifactorial, including concerns with regards to costs and required experience, one of the main arguments has been the uncertainty on whether the risks associated with thrombolysis indeed result in better outcomes in the long-term. In this study, we have shown that not only patients treated with intravenous alteplase have better BI and FAI scores at 5 years but that they also experience a lower mortality risk, with relatively low numbers needed to treat to prevent a death at 10 years. Furthermore, this study was done using data from a real-world setting from a diverse population, and thus our results are generalizable. These findings should provide much-needed evidence to reassure treating clinicians and patients about the long-term benefits of intravenous thrombolysis therapy with alteplase following currently accepted guidelines.

## Acknowledgments

We thank patients, their families, and the fieldworkers who have collected data for the South London Stroke Register since 1995.

## Sources of Funding

We would like to acknowledge the support and funding from the National Institute for Health Research (NIHR) Collaboration for Leadership in Applied Health Research and Care South London at King’s College Hospital NHS Foundation Trust and the Royal College of Physicians, as well as the support from the NIHR Biomedical Research Centre based at Guy’s and St Thomas’ NHS Foundation Trust and King’s College London.

## Disclosures

None.

## Supplementary Material
