# Supplementary material for: Long-Term Survival After Intravenous Thrombolysis for Ischemic Stroke: A Propensity Score-Matched Cohort With up to 10-Year Follow-Up
Source: Stroke. 2018 Feb 12;49(3):607–13. doi: 10.1161/STROKEAHA.117.019889 (PMC5839705; doi:10.1161/STROKEAHA.117.019889)
Supplement: Supplementary file 1 [file str-49-607-s001.pdf]

Supplemental Material

Long-term survival after IV thrombolysis for ischemic stroke: A propensity score-matched cohort with up to 10-year follow-up.

Authors: Walter Muruet[1], MBBS; Anthony Rudd[1], FRCP; Charles DA Wolfe[1], MD; Abdel Douiri[1], PhD.

1 From the School of School of Population Health & Environmental Sciences, King's College London, London, United Kingdom (W.M., A.R., C.D.A.W., A.D.); NIHR Comprehensive Biomedical Research Center, Guy's and St Thomas' NHS Foundation Trust, and King's College London, London, United Kingdom (A.R., C.D.A.W., A.D.); and National Institute of Health Research Collaboration for Leadership in Applied Health Research and Care (CLAHRC) South London, United Kingdom (C.D.A.W.)

Address for corresponding author (Dr. Walter Muruet):

5th Floor Addison House, Guy's Campus King's College London,

London SE1 1UL

United Kingdom

Email: walter.muruet_gutierrez@kcl.ac.uk. Phone: +44 (0)207 848 6612

Number of Supplementary Tables: 2
Number of Supplementary Figures: 2

Supplementary Table I. Comparison of baseline characteristics between treated and control participants

	Original Sample				Matched Sample			
	Thrombolysis (n=246)	Controls (n=1472)	Standardized Difference*	P	Thrombolysis (n=246)	Controls (n=492)	Standardized Difference*	p
DEMOGRAPHICS								
Male, n (%)	123 (50.0)	795 (54.0)	0.08	0.272	123 (50.0)	235 (47.8)	0.05	0.621
Age, mean (sd)	68.0 (15.9)	70.0 (15.1)	0.11	0.111	68.0 (15.9)	69.4 (16.4)	0.09	0.241
Age Categories				0.224				0.306
<45, n (%)	25 (10.16)	93 (6.32)	0.15		25 (10.16)	41 (8.33)	0.06	
45-64, n (%)	72 (29.27)	427 (29.01)	0.01		72 (29.27)	144 (29.27)	0.00	
65-74, n (%)	52 (21.14)	331 (22.49)	0.03		52 (21.14)	92 (18.7)	0.06	
75-84, n (%)	64 (26.02)	384 (26.09)	0.00		64 (26.02)	119 (24.19)	0.04	
≥ 85, n (%)	33 (13.41)	237 (16.1)	0.07		33 (13.41)	96 (19.51)	0.15	
Ethnicity				0.756				0.984
White, n (%)	159 (64.6)	924 (62.8)	0.03		159 (64.6)	315 (64.0)	0.01	
Black, n (%)	67 (27.2)	435 (29.6)	0.05		67 (27.2)	137 (27.9)	0.01	
Other, n (%)	20 (8.1)	113 (16.1)	0.01		20 (8.1)	40 (8.1)	0.00	
PRE- STROKE RISK FACTORS								
Hypertension, n (%)	158 (64.2)	1023 (69.5)	0.12	0.100	158 (64.2)	323 (65.7)	0.04	0.628

Diabetes, n (%)	50 (20.3)	351 (23.9)	0.08	0.268	50 (20.3)	104 (21.1)	0.02	0.851
Cholesterol, n (%)	117 (47.6)	665 (45.2)	0.06	0.471	117 (47.6)	232 (47.2)	0.08	0.979
A Fib, n (%)	41 (16.7)	261 (17.7)	0.01	0.913	41 (16.7)	101 (20.5)	0.09	0.296
TIA, n (%)	25 (10.2)	149 (10.1)	0.07	0.015	25 (10.2)	44 (8.9)	0.07	0.668
IHD, n (%)	25 (10.2)	170 (11.6)	0.04	0.003	25 (10.2)	53 (11.4)	0.02	0.878
Smoker, n (%)	27 (11.0)	332 (22.6)	0.04	0.962	27 (11.0)	56 (11.4)	0.08	0.860
Num. of Risk Factors								
Median (IQR)	2 (2)	2 (2)	0.15	0.032	2 (2)	2 (2)	0.04	0.641
Num RF Categories				0.067				0.985
None, n (%)	58 (23.6)	237 (16.1)	0.20		58 (23.6)	111 (22.6)	0.02	
1, n (%)	51 (20.7)	342 (23.2)	0.06		51 (20.7)	100 (20.3)	0.01	
2, n (%)	66 (26.8)	404 (27.5)	0.01		66 (26.8)	133 (27.0)	0.00	
3, n (%)	51 (20.7)	342 (23.2)	0.06		51 (20.7)	102 (20.7)	0.00	
≥4, n (%)	20 (8.1)	147 (10.0)	0.06		20 (8.1)	46 (9.4)	0.04	
PRE-STROKE FUNCTIONAL STATUS								
Barthel, mean (sd)	19.1 (2.6)	18.8 (3.4)	0.12	0.044	19.1 (2.6)	19.1 (2.8)	0.01	0.891
STROKE SEVERITY								
NIHSS, median (IQR)	10 (11)	5 (7)	0.61	<0.001	10 (11)	9 (12)	0.07	0.111

Neurological Deficit †				<0.001				0.118
Minor, n (%)	43 (17.5)	700 (47.6)	0.62		43 (17.5)	78 (15.9)	0.04	
Moderate, n (%)	119 (48.4)	570 (38.7)	0.20		119 (48.4)	279 (56.7)	0.16	
Mod-Severe, n (%)	43 (17.5)	86 (5.8)	0.45		43 (17.5)	60 (12.2)	0.15	
Severe, n (%)	41 (16.7)	116 (7.9)	0.31		41 (16.7)	75 (15.2)	0.04	
Stroke subtype				<0.001				0.815
TACI, n (%)	69 (28.1)	179 (12.2)	0.46		69 (28.1)	123 (25.0)	0.07	
PACI, n (%)	115 (46.8)	589 (40.0)	0.14		115 (46.8)	245 (49.8)	0.06	
LACI, n (%)	34 (13.8)	449 (30.5)	0.37		34 (13.8)	70 (14.2)	0.01	
POCI, n (%)	28 (11.4)	255 (17.3)	0.16		28 (11.4)	54 (11.0)	0.01	
TOAST Classification				<0.001				0.287
LAA, n (%)	39 (15.9)	156 (10.6)	0.17		39 (15.9)	56 (11.38)	0.13	
CE, n (%)	76 (30.9)	326 (22.2)	0.21		76 (30.9)	137 (27.9)	0.07	
SVO, n (%)	37 (15.0)	493 (33.5)	0.40		37 (15.0)	85 (17.3)	0.06	
Other, n (%)	3 (1.2)	21 (1.4)	0.02		3 (1.2)	10 (2.0)	0.06	
Und, n (%)	91 (37.0)	476 (32.3)	0.10		91 (37.0)	204 (41.5)	0.09	
YEAR OF STROKE				<0.001				0.999
2005, n (%)	6 (2.4)	215 (14.6)	0.37		6 (2.4)	9 (1.8)	0.04	

2006, n (%)	12 (4.9)	159 (10.8)	0.20	12 (4.9)	30 (6.1)	0.05
2007, n (%)	23 (9.4)	154 (10.5)	0.04	23 (9.4)	46 (9.35)	0.00
2008, n (%)	32 (13.0)	127 (8.6)	0.15	32 (13.0)	64 (13.01)	0.00
2009, n (%)	26 (10.6)	147 (10.0)	0.02	26 (10.6)	59 (12.0)	0.05
2010, n (%)	34 (13.8)	141 (9.6)	0.14	34 (13.8)	68 (13.8)	0.00
2011, n (%)	19 (7.7)	126 (7.9)	0.03	19 (7.7)	37 (7.5)	0.00
2012, n (%)	30 (12.2)	116 (7.9)	0.16	30 (12.2)	52 (10.6)	0.05
2013, n (%)	25 (10.2)	101 (6.9)	0.13	25 (10.2)	46 (9.4)	0.03
2014, n (%)	25 (10.2)	83 (5.6)	0.19	25 (10.2)	51 (10.4)	0.00
2015, n (%)	14 (5.7)	103 (7)	0.05	14 (5.7)	30 (6.1)	0.02

* A standardized difference (of means) < 0.25 indicates good balance between groups.

† Based on total NIHSS score: Minor (1 to 4), moderate (5 to 15), moderate to severe (16 to 20), severe (≥20)

A fib, atrial fibrillation; NIHSS, National Institute of Health Stroke Scale; TACI, total anterior circulation infarction; PACI, partial anterior circulation infarction; LACI, lacunar infarction; POCI, posterior circulation infarction; LAA, Large artery atherothrombotic; CE, cardioembolic; SVO, small vessel occlusion; Und, undetermined.

Supplementary Table II. Multivariable Cox Regression on survival

	Survival at 5 years			Survival at 10 years		
Predictor	Hazard Ratio	95% CI	p-value	Hazard Ratio	95% CI	p-value
Thrombolysis (yes)	0.72	0.60-0.87	0.003	0.63	0.48-0.82	<0.001
Age (years)	1.03	1.02-1.03	<0.001	1.05	1.04-1.06	<0.001
Barthel Index (point)	0.95	0.92-0.99	0.008	0.95	0.92-0.98	0.003
Pre-stroke anticoagulants (yes)	1.93	1.40-2.67	<0.001	1.85	1.25-2.73	<0.001
NIHSS (point)	1.05	1.04-1.07	<0.001	1.07	1.05-1.09	<0.001
Post-stroke antiplatelets (yes)	0.55	0.46-0.64	<0.001	0.44	0.35-0.56	0.006
Propensity score (point)	0.46	0.26-1.47	0.081	0.50	0.15-1.61	0.650

Hazard ratios are adjusted by the variables shown in the table.

NIHSS, national institute of health stroke scale.

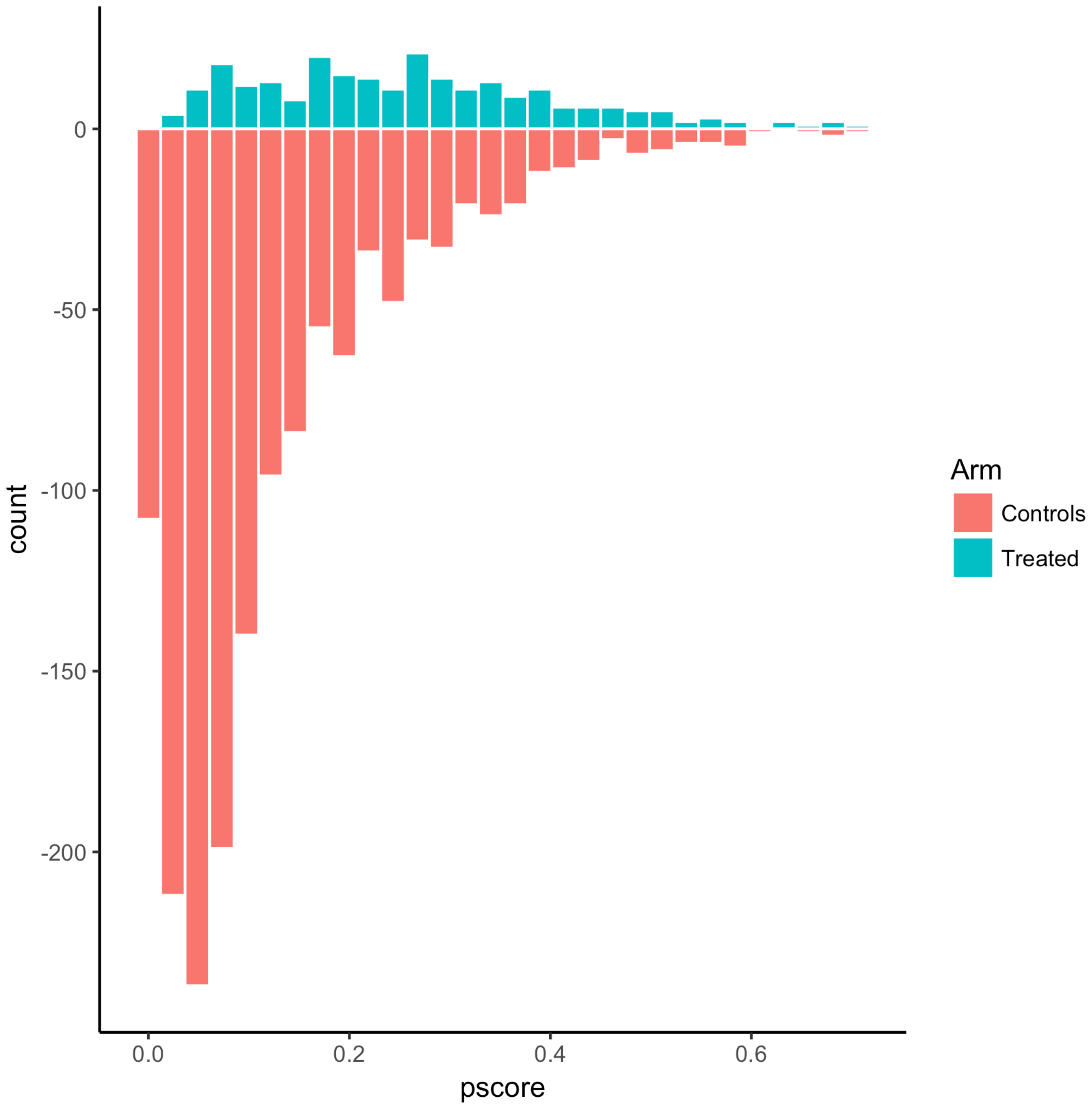

Supplementary Figure I. Distribution of propensity scores between the treated and controls group. An overlap between both distributions suggests good balance between groups.

Predictor	HR	95% CI
Thrombolysis		
Arrival ≤ 3h	0.58	0.40-0.82
Arrival > 3h	1.15	0.55-2.43
Age	1.05	1.04-1.06
Barthel Index	0.94	0.91-0.98
Anticoagulants	1.54	0.91-2.61
NIHSS	1.06	1.04-1.08
Antiplatelets	0.48	0.37-0.63
Propensity Score	1.15	0.54-2.43

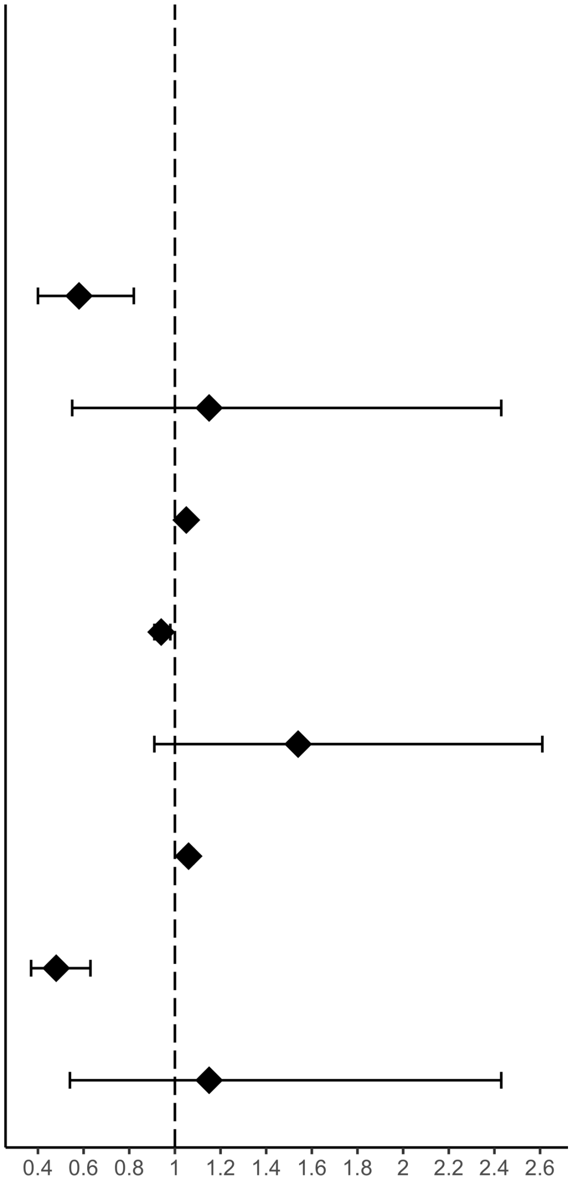

Supplementary Figure II. Forest plot showing the point estimates (Hazard Ratios) with their respective confidence interval for the Cox model on survival at 10 years.
